# Modelling mitral valvular dynamics–current trend and future directions

**DOI:** 10.1002/cnm.2858

**Published:** 2017-02-16

**Authors:** Hao Gao, Nan Qi, Liuyang Feng, Xingshuang Ma, Mark Danton, Colin Berry, Xiaoyu Luo

**Affiliations:** ^1^ School of Mathematics and Statistics University of Glasgow UK; ^2^ Bioengineering College Chongqing University China; ^3^ Department of Cardiac Surgery Royal Hospital for Children Glasgow UK; ^4^ Institute of Cardiovascular and Medical Sciences University of Glasgow UK

**Keywords:** fluid‐structure interaction, left ventricle, mitral valve, numerical methods, soft tissue

## Abstract

Dysfunction of mitral valve causes morbidity and premature mortality and remains a leading medical problem worldwide. Computational modelling aims to understand the biomechanics of human mitral valve and could lead to the development of new treatment, prevention and diagnosis of mitral valve diseases. Compared with the aortic valve, the mitral valve has been much less studied owing to its highly complex structure and strong interaction with the blood flow and the ventricles. However, the interest in mitral valve modelling is growing, and the sophistication level is increasing with the advanced development of computational technology and imaging tools. This review summarises the state‐of‐the‐art modelling of the mitral valve, including static and dynamics models, models with fluid‐structure interaction, and models with the left ventricle interaction. Challenges and future directions are also discussed.

## 1 MITRAL VALVE BIOMECHANICS

**Figure 1:**
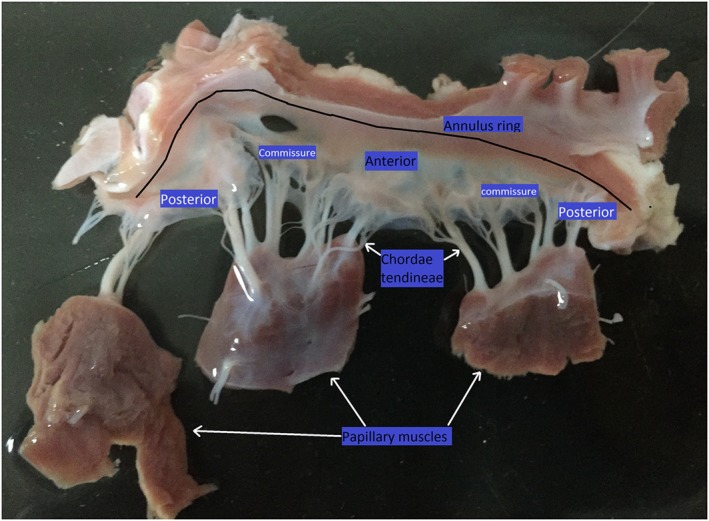
Mitral valve (MV) anatomy from a freshly excised swine heart at our Chongqing Lab, the MV was placed on a flat surface

The mitral valve (MV) is so called because of its shape‐like characteristics to that of a Bishops “Mitre,” a ceremonial headdress wore during traditional Christian ceremonies. The valve is situated between the left atrium and ventricle and functions to allow unidirectional blood flow from the atrium to the ventricle during diastole. Of the two left‐sided heart valves, the other being the aortic valve, the MV has the larger surface area and bears the highest transvalvular pressure difference during systole.^1^ It has a complex structure, consisting of 2 geometrically distinct flexible leaflets, mitral annulus, and the sub‐valvular apparatus comprising the chordal tendineae. Fibrous chords connect the valve leaflets to the papillary muscles, which attach to the free wall of the left ventricle (LV) in anterior‐lateral and posterior‐medial positions. Figure 1 shows the MV anatomy from a freshly excised porcine heart placed on a flat surface.

Dysfunction of the MV includes valve stenosis (defined by variable obstruction to antegrade mitral flow during diastolic LV filling) and regurgitation (in which a proportion of the stroke volume passes retrogradely from LV to left atrium during systole). Mitral valve dysfunction is a common cardiac valvular lesion and remains a major medical problem worldwide.^2^ Mitral regurgitation, for example, can affect up to 5% of the population.^3^ Mitral valve dysfunction can occur as a primary issue related to the mitral valve complex, e.g., Barlows Disease or Rheumatic infection, or secondary to ventricular pathologies, including myocardial ischemia infarction and cardiomyopathy. The significance of MV dysfunction, such as MV regurgitation, is that it is associated with LV and atrial volume overload, chamber dilation with later clinical consequences of atrial fibrillation, and heart failure.

It is reported that as many as 1 in 20 people have some degree of MV prolapse,^4^, ^5^ which will result in mitral regurgitation eventually. The most common cause of mitral valve prolapse is because of chordal fibro‐elastic degeneration with elongation and rupture of the affected chords. Other aetiologies include rupture or dysfunction of the papillary muscles, rupture of chordal tendineae due to infective endocarditis, or abnormal LV wall motion in the setting of myocardial ischemia and primary myocardial disease. Studies have shown that the presence of mitral regurgitation following myocardial infarction doubles the risk of death of post myocardial infarction.^6^ A recent clinical investigation^7^ also revealed that for congestive heart failure with 1 single comorbidity, the most common aetiology is heart valvular diseases.

There are numerous possible treatment options regarding “who, when, and how,” but the management of the individual patients remains difficult. For example, the long‐term benefits of adding mitral valve repair along with coronary‐artery bypass grafting for moderate ischemic regurgitation remains controversial.^8^ This controversy is based in part on the lack of clinical data. Computational modelling of human MV function could lead to new understanding of MV physiology informing mechanisms of failure, new treatment, prevention, and diagnosis. Any disease state resulting in altered stress within the valve components can influence MV disease progression or repair durability.^9^, ^10^, ^11^ However, measuring stress patterns within the MV is extremely challenging. Computational models can fill in this gap by providing detailed deformation and stress patterns.^1^, ^12^, ^13^, ^14^

Compared with the aortic valve, MV has been much less studied owing to its highly complex structure and its coupled interaction with the blood flow and ventricles. Despite this, there is an increasing interest in MV modelling due in part to the advancements in computational technology and cardiac imaging. Various computational models of MV have been developed, starting from simple 2‐dimensional approximation^15^ to 3‐dimensional structure‐only models,^16^, ^17^ and to fully coupled fluid‐structure interaction (FSI) systems.^18^, ^19^ Some of these models were focused on physiological mitral function,^20^, ^21^ others on valvular diseases,^22^, ^23^, ^24^ and surgical interventions.^23^, ^25^, ^26^

The MV models can be broadly grouped into 2 categories: (1) the structure‐only models and (2) the FSI models. The structure‐only models are simpler and are useful for studying the MV closure at physiological and pathological conditions. The FSI models can capture the full MV dynamics throughout the heart beat and allow more realistic boundary conditions to be imposed. In particular, the FSI models can estimate the flow across the MV, which is a strong indicator of the MV function. However, the FSI models are computationally more intensive. Therefore, one has to choose a model suitable for the clinical questions at hand.

Although a number of review papers have been published, these were focused on different angles, such as the general heart valves,^1^, ^27^, ^28^, ^29^ multiscale modelling,^30^ patient‐specific modelling towards clinical translation,^13^, ^14^ valvular intervention,^31^, ^32^, ^33^ valvular engineering,^29^, ^34^ and fluid dynamics modelling of valvular mechanics.^35^, ^36^ This review summarises the most recent MV modelling with an emphasis on FSI, the interaction with the heart, and challenges and future directions.

## 2 STRUCTURE‐ONLY MV MODELS

Biomechanical studies of the MV using structure‐only finite element (FE) models have been performed by a number of groups since the 1990s. Kunzelman and coworkers were the first to use a 3D‐FE approach to simulate mitral function,^17^, ^20^ to explore the biomechanics underlying valvular diseases,^22^, ^23^, ^37^ and to evaluate surgical corrections.^23^, ^25^, ^38^ Lim et al^39^ investigated the asymmetric stress pattern in the MV using a 3D dynamic ovine MV model. In a series of studies, Prot et al^40^, ^41^, ^42^, ^43^ investigated the mitral behaviour using a transversely isotropic strain‐energy function. Their model was used to predict the mechanical difference in MV function of a healthy heart compared with MV function in a situation of hypertrophic obstructive cardiomyopathic heart.^24^ They observed that including the active muscle contraction reduced the non‐physiological bulging of the modelled leaflet.^41^, ^43^ Weinberg and Kaazempur‐Mofrad^44^ treated the embedded collagen fibre as the 5th degree of freedom at each node and studied the large‐deformation and anisotropic behaviours of the valvular leaflets. MV surgical interventions were also modelled^38^, ^45^ to study the effects of annuloplasty procedures,^23^, ^46^, ^47^, ^48^, ^49^, ^50^ the biomechanical response to the Alfieri stitch technique,^51^ mitral annular contraction,^52^ and neochordal replacement and implantation.^25^, ^53^

In this section, we briefly review the MV modelling in the categories of model construction, constitutive laws, and validation and verification. Note that these categories are also related to other types of MV modelling, such as FSI modelling.

### 2.1 Image‐based geometry reconstruction

An accurate, fast and reliable MV model reconstruction based on in vivo imaging data is the first step in the MV modelling aimed for clinical translations. The complex MV apparatus, such as the thin leaflets, the chordae tendineae, and the large leaflet motion, makes it difficult to acquire the clinical images because of the constrained imaging modalities regarding the temporal/spatial resolutions, quality, and reproducibility. Earlier MV modelling studies assumed that the valve is symmetrical to the plane perpendicular to the valve orifice and that the annulus is circular.^17^, ^22^, ^23^, ^26^, ^54^, ^55^ Recent studies started to take advantage of fast‐developing clinical‐imaging modalities,^56^ including echocardiography‐ 2D and 3D (ECHO), computed tomography (CT), and cardiac magnetic resonance imaging (MRI). In particular, the 3D+t transesophageal echocardiography (TEE) is commonly used for MV function evaluation in the clinic. Various MV geometry reconstructions were based on imaging derived from ECHO,^57^ CT,^16^, ^58^ and MRI.^18^, ^59^ However, it is often difficult to image in detail the sub‐valvular structures either with ECHO or MRI, especially the chordae tendineae. Therefore, sub‐valvular apparatus was often described in a generalised anatomical way in models derived from ECHO or MRI data.^18^, ^59^ CT can increase the level of anatomical detail of the subvalvular apparatus, but the patient is exposed to radiation. Wang et al^11^ reported a patient‐specific FE model of a healthy human MV reconstructed from multi‐slice CT scans with detailed leaflet thickness, chordal information, and mitral annulus dynamic motion. Micro‐CT is another widely used modality that can provide detailed MV leaflets and chordal structure for in vitro/ex vivo MV imaging but is usually unavailable with in vivo imaging when using ECHO and MRI.^60^, ^61^, ^62^ Manually constructing MV geometry from limited imaging data is not an optimal way, because it is highly dependent on the operators' experience, and usually associated with low reproducibility and reliability. Automatic and faster imaging processing methods are being developed for MV geometry reconstruction^63^ in the recent years. Schneider et al^64^, ^65^ developed an automatic pipeline for MV geometry delineation from 3D+t TEE images. By using machine learning to detect the MV on 3D TEE or CT data, Ionases et al.^66^ developed fast and accurate methods to estimate the MV dynamics. Mansi^63^ further developed an integrated framework for MV reconstruction of 25 patients from TEE images.

### 2.2 Material models

Knowledge of MV material characterisation is essential in understanding the MV function and in designing and evaluating new surgical repair procedures.^67^ The MV leaflets and its sub‐apparatus, like many other soft tissues, are anisotropic, highly nonlinear, and heterogeneous. This is a result of the complex underlined microstructures, in particular, the highly organised collagen fibres, spanning circumferentially with regional and transmembrane variations.^68^ It is challenging to model the mechanical behaviour of the MV accounting for all the anisotropic and nonlinear behaviour; the coupling between different directions, the differences of in vivo and ex vivo, and the residual strain and strain‐path dependence. The MV material properties are mainly derived from animal heart valvular experiments^17^, ^69^, ^70^, ^71^, ^72^, ^73^, ^74^, ^75^, ^76^. In uniaxial studies of excised leaflets, Kunzelman and Cochran^17^ measured the stress‐strain behaviour of MV within distinct pretransitional and posttransitional regions. In biaxial studies of excised anterior leaflets, May Newman and Yin^12^, ^69^ found that the pretransitional stiffness values ranged from 0.06 to 0.09 *N*/*m* *m* ^2^, and posttransitional stiffness values from 2 to 9 *N*/*m* *m* ^2^. These leaflet data were commonly implemented in the hyperelastic FE analyses.^19^, ^41^, ^77^ Sacks et al^75^ used a left heart simulator and graphite markers to study the in vitro surface strains in the porcine anterior mitral valve leaflets and demonstrated a nonlinear relationship between transmitral pressures and leaflet strains. Ex vivo mechanical tests on porcine and human mitral apparatus by Prot and colleagues^24^, ^40^, ^42^, ^43^, and biaxial tests on human mitral leaflets by Wang and co‐workers,^16^, ^78^ all suggested that MV leaflets are highly nonlinear and anisotropic. These properties are represented using either the Fung‐type strain energy function or the strain invariant‐based fibre‐reinforced strain energy function.

However, studies have found that in vivo valvular properties of MV are very different from the material response ex vivo.^79^ To overcome this challenge, several studies have attempted to acquire data from in vivo animal experiments combined with an inverse FE analysis and invasive surgical operations. The first attempt at quantifying the in vivo leaflet strains was from Sacks et al,^74^ who used a sonomicrometry transducer array. They found a similar nonlinear relationship between pressure and leaflet strains in ovine anterior MV leaflets.

Krishnamurthy et al^79^ estimated the mechanical response, albeit with a linear isotropic material model, of the anterior mitral leaflets by sewing radiopaque markers to MV in a sheep. They found that the moduli of the ovine MV anterior leaflet estimated in vivo from inverse FE analysis were much greater than those reported from in vitro studies, and suggested that it resulted from activated neurally controlled contractile tissue within the leaflet that is inactive in excised tissues.^67^ However, there was another explanation suggested by Amini et al:^80^ the existence of prestrain. In an animal study, they demonstrated that there exist substantial in vivo residual strains/stresses in the leaflets.^80^ Later, Rausch et al^68^ found that by including 30% prestrain (residual strain), they could match both in vivo and ex vivo experimental uniaxial stress‐stretch data using 2 different anisotropic constitutive laws, and the estimated parameters were highly sensitive to the prestrain level. Further studies are needed to clarify the potential roles of the residual strain/stress and active stiffening in the MV.

Hyperelastic constitutive material models are commonly used in MV modelling to account for subject‐specific anisotropic behaviours of the MV and its sub‐apparatus. These constitutive laws can be broadly classified into 2 categories: Fung‐type strain energy function; and strain invariant‐based fibre‐reinforced strain energy function. Lee et al^81^ inversely estimated the in vivo material properties of ovine MV anterior leaflets with various nonlinear anisotropic hyperelastic constitutive laws, and they concluded that the transversely isotropic law produced the most accurate results. Still, there is no definite answer to the question, which strain energy functions should be used. Novel mechanical experiments and constitutive laws at different structural scales may need to be developed to best characterise the mechanical responses of the MV.

### 2.3 Validation and verification

Despite substantial challenges, a growing effort has been made in validating and verifying the computational cardiovascular models. Some were achieved through comparisons to experimental benchmark data,^82^ clinical images,^21^, ^43^, ^59^, ^63^, and independent computational models.^83^ The U.S. Food and Drug Administration conducted an initial study to compare flow predictions from computational fluid dynamics in an idealised cardiovascular device to experimentally measured data.^82^ Other benchmark studies in cardiovascular modelling can be found in.^84^, ^85^, ^86^ Mitral valve models are mainly validated by comparisons with in vitro or ex vivo experiments,^60^, ^61^, ^62^, ^87^, ^88^ and partially with clinical imaging.^43^, ^59^ It is important to report stress and strain data in all the MV models to facilitate validation and verification. In Table 1, we summarise the stress and strain data from a number of publications, averaged from some typical regions of MV (trigons, the belly of the anterior leaflet). Table 1 shows that we still have a long way to go to identify the coherent stress and strain patterns from different experimental and numerical studies. For example, the stress reported by Krishnamurthy et al^79^ was an order higher than other studies. Relevant results, such as the maximum strain of the MV, stress in the anterior leaflet, and papillary muscle forces, were not always reported. In general, there is a lack of experimental and computational benchmark data for the MV models. Direct and thorough validations with clinical imaging data are largely unreported because of (1) the over‐simplifications of developed models, (2) limited clinical data, which are often of poor resolution, and (3) unknown material properties and boundary conditions.

**Table 1:** Stress/strain data published from various experimental and numerical studies.

				strain in AML belly		peak pressure
**Experiments**	in vivo/in vitro	max strain		(N)	PM forces	(mmHg)
Jimenez et al.^99^	in vitro			0.11 ± 0.049^*c*^		120
				0.22 ± 0.07^*r*^
Rausch et al.^100^	in vivo	0.13 ± 0.047^ + ^		0.05 ± 0.027^*c*^		97.2 ± 7.8
				0.078 ± 0.043^*r*^
Sacks et al.^74^	in vivo			0.025 ∼ 0.033^*c*^		90 ∼ 200
				0.16 ± 0.20^*r*^
Sacks et al.^75^	in vitro			0.1^*c*^		120
				≈0.3^*r*^
He et al.^73^	in vitro	0.025 ∼ 0.1(PML)^*c*^				120
		0.2 ∼ 0.4(PML)^*r*^
Padala et al.^101^	in vitro (20% saddle)			0.1 ± 0.08^*c*^		120
				0.29 ± 0.08^*r*^
	max stress		stress in AML belly		max PM forces	peak pressure
**Models**	(kPa)	max strain	(kPa)	strain in AML belly	(N)	(mmHg)
Wang et al.^16^	334.0 (AML)^ + ^		160^ + ^	0.1^*c*^ 0.3 ^*r*^	4.51 (ALP), 5.17 (PMP)	110
	251.9 (PML)^ + ^					
Gao et al.^18^			142^∗^	0.02^∗^	3.0, 3.34	150
Prot et al.^42^	386 (AML)*†*				3.97	120
	243 (PML)^*†*^
Kunzelman et al.^19^	224 (AML)^ + ^	0.17 ∼− 0.51^*c*^			2.6	95
		0.04 ∼ 0.25^*r*^
Kunzelman et al.^102^	100 ∼ 410(AML)^+^					120
	8 ∼ 225(PML)^ + ^
Toma et al.^60^	1 ∼ 1000^ + ^				2.6	100
Lau et al.^103^	566 ∼ 635(PML)^ + ^					120
Wenk et al.^104^	85.4(AML average) ^*†*^		119 ^*†*^			91.46
	72.9(PML average)*†*
Votta et al.^54^	396(AML)^ + ^					120
	194(PML)^ + ^
Votta et al.^77^	550(AML)^ + ^			0.11 ∼ 0.15^*c*^,	4 ∼ 6.5	120
				0.29 ∼ 0.41^*r*^
Stevanella et al.^52^	300(AML)^ + ^	0.18(AML)^*c*^			4.11	120
	100(PML)^ + ^	0.56(AML)^*r*^
		0.08(PML)^*c*^
		0.46(PML)^*r*^
Stevanella et al.^105^	430(AML)^ + ^			0.13 ∼ 0.16^*c*^	6.11(ALP), 6.92(PMP)	120
	120(PML)^ + ^			0.25 ∼ 0.3^*r*^
Dal Pan et al.^51^	330(AML)^*†*^					120
	252(PML)^*†*^					
Krishnamurthy et al.^79^			1540 ± 838^*c*^	0.042 ± 0.006^*c*^		60 ∼ 70
			1512 ± 826^*r*^	0.13 ± 0.05^*r*^		
Lee et al.^81^			432.6 ± 46.5^*c*^	0.11^*c*^		90
			241.4 ± 40.5^*r*^	0.32^*r*^		
Rim et al.^106^	700 ∼ 900				3.3 ± 0.6	≈100

+: principle stress/strain;

*: fibre(circumferential) stress/strain;

†: Von Mise stress/strain.

AML: anterior mitral leaflet; PML: posterior mitral leaflet; PM: papillary muscle; ALP: anterolateral papillary muscle; PMP: posteromedial papillary muscle.

*c*: circumferential direction; *r*: radial direction.

## 3 MODELLING MV WITH FLUID‐STRUCTURE INTERACTION

### 3.1 An overview of FSI methodology

The function of MV is driven by the transvalvular pressure difference between the left atrium and the LV as well as the ventricular dynamics resulting from the well coordinated myocardial contraction and relaxation. In the structure‐only models, these are usually approximated by a homogeneous pressure load applied onto the leaflet surface. Although this approximation is reasonable when simulating the fully opened or closed configurations, studies have pointed out that the flow patterns inside the LV have important effects on MV functions. For example, it has been shown that vortex formation helps the MV closure at end‐diastole.^89^

The challenge of FSI modelling is that the fluid mechanics is most conveniently described using the Eulerian formulation, while the solid equations are normally described in the Lagrangian formulation.^90^ In addition, there are large deformations associated with abrupt MV opening and closure, together with the complex dynamics of the sub‐valvular apparatus. General FSI methodology can be categorised into 2 groups: boundary‐conforming methods and nonconforming boundary methods, although the application of these methods to the MV modelling is still in its infancy.

#### 3.1.1 Boundary‐conforming methods

In the boundary‐conforming methods, there are 2 sets of different nonoverlapping meshes and variables for the fluid and solid domains. In the fluid‐structure interface, displacement and stress continuities are typically constrained. One of the most widely used approaches to constructing such models is the Arbitrary‐Lagrangian‐Eulerian formulation (ALE),^91^ in which the computational fluid grid is fitted to and deforms with the moving structural boundary. The ALE formulation has been mostly applied to simulate the flow through mechanical heart valves.^92^, ^93^, ^94^ This type of FSI approach requires dynamic mesh generation when large structural deformations are present, thus substantially complicates the implementation of the scheme.^95^ For example, Cheng et al^96^ constructed the intermediate mesh through interpolation from previously generated meshes and followed by a smoothing process to obtain the final mesh. The difficulties with the ALE methods are further exacerbated in problems involving large structural displacements, as is the case of valvular leaflets. In such problems, obtaining smooth and well‐conditioned computational meshes at every time step can be far from trivial, and frequent re‐meshing may be the only option. It is for this reason that the ALE approaches are mostly developed to address problems with relatively simple geometry and moderate deformations, such as flow in arteries.^97^, ^98^ To the best of our knowledge, applications of the ALE methods to MV dynamics have not been reported.

#### 3.1.2 Nonconforming boundary methods

Nonconforming boundary methods provide alternative ways to model FSI. In these methods, the fluid domain is described in Eulerian form with either structured or unstructured mesh, and the solid is discretised in the Lagrangian form either with a set of the Lagrangian nodes or finite element meshes. The fluid and solid meshes can be independently generated; the effects of the solid are accounted by incorporating an explicit/implicit body force to the momentum equations. The fluid and structural dynamics can be solved using either partitioned approach or monolithic approach. Nonconforming boundary methods remove the need for dynamic mesh generation, thus are extremely well‐suited for cardiovascular applications that involve large structural deformations. Nonconforming boundary methods can be further classified as (1) diffused interface approach, such as the immersed boundary method^107^ and the fictitious domain methods^95^, ^108^ and (2) non‐diffused interface approach, such as the sharp‐interface method.^109^

The classical immersed boundary (IB) method pioneered by Peskin in the 1970s^107^ is the earliest work of the overlapping method for heart valves and has since been widely used to simulate the dynamics of the heart and its valves.^59^, ^110^, ^111^, ^112^, ^113^, ^114^, ^115^, ^116^ In the IB framework, the momentum and incompressibility of the whole coupled system are formulated in the Eulerian frame of reference, and the structural dynamics (motion, forces) are described in the Lagrangian frame of reference. Smoothed Dirac delta function kernels are used to couple the Eulerian and Lagrangian variables.^95^, ^112^, ^117^, ^118^ The structural models are usually described using systems of elastic fibres.

Methods combining IB with FE are recent extensions of IB methods that use the finite‐element–based structural models^119^, ^120^ to make full use of hyperelastic constitutive laws. One of these is the hybrid finite difference‐finite element IB method (IB/FE) developed by Griffth and Luo,^120^ which is built within the framework of adaptive and distributed‐memory parallel implementation of the immersed boundary method (IBAMR) *
https://github.com/IBAMR/IBAMR
, has significantly increased the accuracy and flexibility of classical IB methods and is well verified against ALE solvers^115^ and other FE packages^85^, ^121^ at steady flow conditions. This approach has been successfully applied to modelling MV dynamics.^18^

The fictitious domain (FD) method, first developed by Glowinski et al,^108^ is another widely adopted approach for cardiovascular FSI problems.^90^ Similar to the IB approach, the FD method couples the fluid and solid domains using a Lagrangian multiplier to constrain the motion of the fluid‐structure interface. Unlike the IB approach, the FD method solves both the fluid and solid momentum equations. It has been successfully applied to simulate flow in 2‐dimensional and 3‐dimensional tri‐leaflet heart valves.^122^, ^123^, ^124^ Van Loon et al^125^, ^126^ pointed out that one issue of the FD method is that it cannot yield accurate results for the viscous shear stresses on the solid boundary and proposed a combination of the FD method with adaptive mesh refinement to resolve this issue. A combined FD method and ALE was developed by Van de Vosse et al^127^ to simulate valvular dynamics in a simple left‐ventricular flow model. Recently, by implementing FD within a coupled ALE fluid‐structure framework, McCormick et al^128^, ^129^ investigated the LV function under assist device support. The commercial explicit solver LS‐DYNA (Livermore Software Technology Corporation, Livermore, CA, USA) uses similar approach as the FD method and has been used to simulate MV FSI problems.^19^, ^28^ Kamensky et al^130^ introduced an immersogenometric variational framework, following the FD approach developed by Baaijens,^90^ to model bioprosthetic heart valves under physiological conditions, in which the NURBS surface representation of heart valves was directly immersed in a background fluid mesh. Hsu et al^131^ presented a comparison between FSI analysis using immersogenometric variational framework and structural‐only simulation. They found that valvular leaflet deformation from their FSI modelling is in a better physiological realism compared to the structure‐only model.

The sharp interface IB method was introduced to overcome the difficulty in the aforementioned IB and FD approaches, i.e., the approximation of the discrete Delta function leads to the smeared/diffused fluid‐structural interface.^95^ A class of sharp‐interface immersed boundary methods was developed. Examples are the cut‐cell methods^132^ and the curvilinear immersed boundary method developed by Sotiropoulos's group.^109^, ^133^ Blood flow has been successfully simulated using the curvilinear‐immersed boundary method in aortic valves under physiological conditions.^134^, ^135^, ^136^ Vigomostad et al^137^ introduced a partitioned approach to couple the fluid and solid solvers and used an immersed sharp interface for the fluid‐structure interface. Using this method, they simulated MV dynamics under physiological flow Reynolds numbers and realistic material properties.

Other nonconforming boundary methods include the combination of the IB method with the lattice Boltzmann method^138^ and smoothed particle hydrodynamics.^139^ In the former, the leaflets are treated as fibres and the fluid domain is decomposed into a set of lattice nodes. In the latter, the fluid is described as a set of particles, and a penalty‐based contact scheme is used to model the interaction between the fluid particles and the solid. The solid can be described using the FE methods.^60^, ^140^

### 3.2 Fluid‐structure interaction models applied to the MV

Using the classical IB method, McQueen and Peskin simulated the MV dynamics in a contractile LV in the 1980s.^141^, ^142^, ^143^ Parallel implementation of the IB models was developed on a shared memory machine^144^ to simulate the blood flow in a beating mammalian heart with 4 ventricles, mitral, aortic, tricuspid, and pulmonary valves, all of which were represented by bunches of elastic fibres. Contractile elastic fibres were used to represent cardiac muscles. Contraction and relaxation of elastic fibres were simulated by changing elastic parameters at an appropriate time. Tay et al^145^ recently compared the simulated results from the model developed by McQueen and Peskin^144^ with 4D MRI‐flow measurements. Although the classical IB model was based on highly simplified soft tissue structure, it provided some agreement of hemodynamics with recorded MRI.

Einstein, Kunzelman, and coworkers were the first to use a fluid‐coupled 3D computational model to simulate normal and pathological mitral function.^19^, ^146^, ^147^ They used the LS‐DYNA and finite element discretization for the MV. The leaflet material was described as an oriented Gaussian‐distributed population of embedded collagen fibres in an isotropic gel‐like matrix;^148^ material parameters were determined from published biaxial experiments by May‐Newman and Yin.^12^ The chordae were assumed as stretch‐only cables with nonlinear stress/strain relationship. By constructing a symmetric MV model from porcine experimental data, Einstein et al^146^ examined the generation of the first heart sound. These material models are more physiologically realistic compared to the fibre representations in the classical IB method.^59^, ^143^, ^144^ Their results confirmed that changing of the anisotropy of the valvular properties can profoundly alter the valvular function and increased MV stiffness can lead to changes in the peak frequency of heart sounds.^19^, ^28^, ^147^

Lau et al^103^ used LS‐DYNA to compare MV dynamics with and without FSI, based on linear material models. They bathed the MV model in a straight tube and a U‐shaped ventricular volume. Their results suggested that the stress patterns during the closure were similar for all models, but the discrepancies between the structure‐only and the FSI models are due to the difference in the applied pressure. Moreover, the U‐shaped ventricular‐like volume generated slower fluid velocity with increased vorticity compared to the tubular geometry. They further investigated the edge‐to‐edge repair technique^26^ and found that after a repair, the maximum principle stress in diastole was 200% greater than in that of a normal MV and that there was 44%‐50% reduction in the peak‐flow rate. Recently, Toma^60^ developed and validated an FSI MV model using an in vitro ovine MV system. Smooth particle hydrodynamics was used to model the FSI dynamics. Material parameters were tuned by matching the measured MV dynamics. Chordal and papillary muscle forces were examined from the simulations, and good agreements were found between the computational model and in vitro force measurements.

Using a 2D‐MV model together with the left atrium and ventricle model derived from echo data, Dahl et al^15^ studied MV behaviour during the diastolic filling. Fluid‐structure interaction simulation was conducted using the FD approach. They concluded that the asymmetric leaflet geometry was important for accurately predicting the MV flow pattern. A prototype of computational modelling of MV FSI dynamics during diastole mounted in a simplified LV geometry was reported by Chandran et al^32^ using a sharp interface IB framework.^137^ In this model, FEAP^149^ was used for the structural solver, and experimentally derived nonlinear anisotropic material properties were adopted for the leaflets.

On the basis of the classical IB methods,^150^ a full three‐dimensional FSI model of a polyurethane bioprosthetic MV has been studied by the authors' group in a series of studies.^89^, ^113^, ^115^, ^151^, ^152^ The elastic properties of the modelled valve (including the valve leaflets and the artificial chordal tendineae) were represented by a system of “pseudo elastic fibres" as in,^144^ the elastic properties of these fibres were determined from the material properties of the prosthetic valve. The simulations successfully predicted the dynamic performance of the prosthetic mitral design. Yin et al^89^ investigated the same prosthetic chorded MV inside a prescribed moving LV geometry derived from MR images. They found that when the MV was placed inside a dynamic LV, the flow field was strongly asymmetric; the stretches in the commissural region were also higher than that of other areas. Griffith et al^153^ found that both the valve opening and closure were more accurately represented using an adaptive IB method. Better MV closure and agreement with experiment measurements were achieved when the bending stiffness of the mitral leaflets was modelled.^115^ Ma et al^59^ constructed a human MV FSI model based on in vivo MRI and simulated the MV dynamics under physiological transvalvular pressure loads. The computed opening shape and flow rates agreed well with the corresponding in vivo measurements, and the results suggested that subject‐specific MV geometry can have a significant influence on the predicted MV behaviour. One major limitation of those studies is that the discrete pseudo elastic fibres cannot model the anisotropic hyperelastic behaviour of the MV properly in large deformation.^16^, ^154^ Recently, Gao et al^18^ extended the human MV model^59^ to a transversely anisotropic, hyperelastic material using the IB/FE framework.^120^ The model results agreed well with the measured MV opening and closure, as well as the transvalvular flow rate. Predicted stress and strain patterns were comparable to published structure‐only MV models at the fully closed state.^16^

## 4 COUPLED MV‐LV MODELLING (WITH/WITHOUT FSI)

In most of the current MV modelling, simplified boundary conditions are used. For example, nodal displacements are imposed to mimic motions of papillary muscles and mitral annulus ring;^16^, ^42^, ^52^ homogeneous pressure loading is used to represent the dynamic blood‐valvular interactions.^16^, ^24^, ^54^, ^61^, ^62^, ^77^, ^102^ Even in the FSI models, the MV and its sub‐apparatus are usually mounted into a tube‐like or ventricular‐like shapes, with pressure or flow waveforms applied at the inflow and outflow tracts.^18^, ^26^, ^32^, ^59^, ^60^, ^103^, ^115^, ^146^ More sophisticated boundary conditions have also been introduced to account for the ventricular dynamics, using either a prescribed motion^89^ or a coupled MV‐ventricular model.^104^, ^155^

Despite the strong coupling between the MV and LV, few modelling studies have integrated the MV and LV simultaneously into a single model,^104^, ^156^, ^157^ particularly with FSI.^144^ This is because integrated MV‐LV approach faces a number of challenges:

- the geometries of the MV and sub‐valvular apparatus are complex;
- MV dynamics is affected by chordae that are connected to the papillary muscles, which are embedded in the LV
- modelling of a contracting LV itself is nontrivial;^158^, ^159^
- the mitral annulus is nonplanar and dynamic;
- MV leaflets have large deformation strongly coupled with ventricular flow; and
- parameter inference is difficult because of the lack of data from in vivo measurements.

To our best knowledge, only 2 structure models of MV‐LV dynamics have been reported in the literature. Wenk et al^104^ developed an FE model of the LV, mitral apparatus, and chordal tendineae using the MR images from a sheep with moderate mitral regurgitation after posterior‐basal myocardial infarction. They found that reduced stiffness in the infarct region with increased stress around the chordal connection points would further worsen the MV regurgitation. Using the same model, Wong et al^156^ compared the stress reduction when using a saddle shaped and asymmetric mitral annuloplasty ring. More recently, Baillargeon et al^157^ used a human cardiac function simulator (Dassault Systemes's living heart project) to simulate the mitral regurgitation caused by infarction of posterior papillary muscle. They also simulated surgical corrections using a undersized annuloplasty ring. The same simulator was used by Rausch et al^160^ to study the effects of surgically induced tissue strains/stresses with different ring size. The human cardiac function simulator includes the 4 ventricular chambers, valves, major vessels, electrophysiology, and detailed fibrous architecture in the myocardium. However, except of the early work by McQueen and Peskin,^144^ FSI modelling of MV‐LV interaction has not been studied.

**Figure 2:**
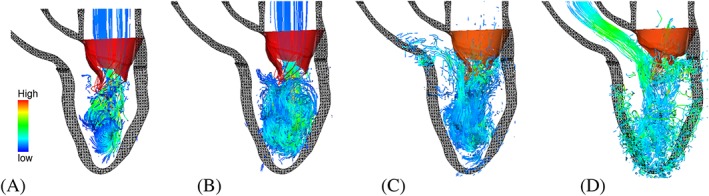
Streamlines in an integrated MV‐LV model^155^ at (A) early diastolic filling, (B) late diastolic filling, (C) when the MV is closing, and (D) middle of the systolic ejection. Coloured by the velocity magnitude. LV indicates left ventricle; MV, mitral valve

An integrated MV‐LV model with FSI has been developed by the authors' group.^155^ This was the first 3D FSI MV‐LV model that was based on MR images and included the LV contraction, nonlinear soft tissue mechanics, and FSI within the IB/FE framework. The instantaneous streamlines of this model in Figure 2 show how flow patterns are strongly affected by the LV deformation. Despite several simplifications, results of the MV‐LV model compared favourably with the in vivo imaging.

## 5 CHALLENGES AND FUTURE DIRECTIONS

Current guidelines for the management of patients with MV diseases have been criticised for their lack of treatment recommendations based on randomised and controlled trials.^161^, ^162^ Furthermore, although the clinical consequences of MV disease can be assessed, the mechanical environment is not, and thus computational modelling may provide a unique opportunity to understand MV function and to predict clinical outcomes. Although various MV models are being developed, we have a long way to go before modelling can lead to clinical translations. Here we discuss a number of current challenges in the area and offer our view of future directions.

### 5.1 Model uncertainty

Along with the advancement of clinical imaging and computational techniques, the complexity of MV models is increasing. As we are moving towards to stratified medicine guided by precise predictions of patient‐specific models,^163^ there is an urgent need to estimate the uncertainty arising from how different aspects affect the modelling results. In other words, how confident can we trust our model predictions? There are many uncertainties in the modelling procedure. These include (1) inaccurate experimental measurements; (2) image segmentations errors; (3) errors in geometry reconstruction; (4) model simplifications; (5) choice of material models (linear or nonlinear) and boundary conditions (homogeneous/heterogeneous pressure loads); and (6) unknown internal parameters. Most of the MV papers have not reported the uncertainty of the model results.

Despite these uncertainties, MV models can provide insights unattainable from experiments alone, as George Box once said, “All models are wrong, but some are useful.”^164^ To make the models meaningful, we need to address the model uncertainty in a rational way. Recently, statistics inference, such as Monte Carlo methods^165^ and the Gaussian process emulator,^166^ has been actively applied in many areas to quantify model uncertainty. Such an approach can provide confidence intervals of predictions with probability distributions of model inputs inferred from limited data sets.^167^ The uncertainty and sensitivity analysis of the multi‐scale and multi‐physical MV model may be prohibitive when using the Monte Carlo methods, but the Gaussian process emulator^168^ looks attractive because it can emulate a broad range of models built from a small set of model runs, thus significantly reduces the computational cost. Interest in statistical inference within biomechanical modelling is growing; for example, it is one of the main focuses of the EPSRC Centre of SoftMech †
www.softmech.org
. It is anticipated that with the help of state‐of‐the‐art machine learning methods, we may characterise the uncertainty in the predictive MV modelling.^169^

### 5.2 Boundary conditions and valvular‐heart interaction

One essential component of biomechanical modelling is the choice of proper boundary conditions. The boundary conditions used in most of the current MV models are over simplified, by either ignoring the FSI effects or the ventricular dynamics, or both. To improve the accuracy of patient‐specific modelling, boundary conditions need to be based on in vivo measurements. Flow rate across the MV and AV is now measurable using phase‐contrast MRI. However, obtaining subject‐specific ventricular pressure waveform noninvasively is still a challenge. Imaging the motion of the papillary muscles and the mitral annulus ring is also tricky; CT has higher resolution than MRI but only captures a static image in time, therefore will not be able to clarify motion or geometry changes within the cardiac cycle. ECHO can be used routinely, but its image quality is operator dependant and requires multiple perspective orientations to build a complete analysis. Advances in 3D echo may overcome many of these limitation. The effect of ventricular dynamics on the MV may be modelled by prescribed motion. However, this will not capture the valvular‐ventricular interaction unless ventricular dynamics is fully modelled. Indeed, coupling MV with LV further complicates the choice of the boundary conditions since the valves' actions are controlled by the pump function. The flow across MV needs to be accommodated by the LV expansion during diastole. In fast diastolic filling, the flow across MV results from the LV relaxation. Thus, an accurately modelled myocardial relaxation process is needed along with pressure boundary conditions in the left atrium. In systole, systemic circulation contributes to the physiologically pressure/flow rate boundary conditions, and this may be modelled using either a simplified Windkessel model or 1‐dimensional structure tree model.^170^ Because the flow across AV is driven by the LV contraction, a pressure boundary condition in the AV plane will be easier to implement, and the simulated AV flow rate can be used to validate the model with measured flow rates. Other boundary conditions may also be needed to account for ventricular structure dynamics, such as the motion of the LV basal plane, constrains of the pericardium.

In addition, MV‐induced heart failure involves full interactions not only between the MV and the LV but also the right ventricle and the atria. A coupled valvular‐complete heart model is increasingly recognised as a critical step towards accurately quantifying cardiac function.^157^, ^160^ The interaction between heart valves and the heart chambers requires modelling of the blood flow,^36^ preferably in a 4‐chamber heart. To date, very few studies focused on physiologically realistic valvular‐heart models,^104^, ^157^ even fewer reported valve‐heart modelling with FSI.^144^, ^155^ This is primarily due to the limitations of clinical imaging, lack of computational power, and complexity in multi‐physics modelling. With the fast development of clinical cardiac‐imaging modalities and computational techniques, personalised valvular‐heart models will lead the way for evaluation of MV disease, heart function, and surgical interventions.

### 5.3 Multi‐scale modelling

There is growing evidence that changes in mechanical environments affect functions in all length scales.^1^, ^171^, ^172^ Weinberg and coworkers^173^, ^174^ are among the pioneers to develop multi‐scale models of aortic valves. Different components in MV serve different purposes. For instance, collagen fibres are load bearing, and the valvular interstitial cells (VICs) maintain the integrity of MV structure. Multi‐scale constitutive laws for MV leaflet that integrated collagen fibre networks have attracted much attention, where a Gaussian distribution of collagen fibres is used for describing the collagen fibre stress/strain relationship at tissue level.^62^, ^147^, ^148^ Zhang et al^175^ proposed a meso‐scale constitutive model for MV leaflets by including the contributions from collagen and elastin fibres at different sublayers of MV leaflets and reported excellent agreement between the predicted mechanical behaviour and experiments. Several studies^176^ have tried to quantify VIC deformation to find the biomechanical links to a deleterious valvular structure via remodelling process (protein biosynthesized and enzymatic degradation). In general, it is difficult to estimate personalised MV material parameters from limited clinical data within multiple length scales.^1^, ^30^

Novel mechanical experiments and constitutive laws at different structural scales need to be developed to best characterise MV mechanical responses. Clearly, much more experimental and computational work is required to bridge the gaps between tissue‐ and organ‐level behaviours, cellular mechanotransduction, and mechanobiological adaptations.

### 5.4 Towards patient‐specific modelling for clinical translation

Patient‐specific models can be used to determine medical and surgical strategies for the treatment of valvular heart diseases.^29^ However, the accuracy of MV models is particularly sensitive to the valvular geometry, material properties, and the boundary conditions imposed. Thus, generic models may provide very different results to that of an individual patient. Besides, estimating human MV material properties from in vivo data remains difficult. These are among the grand challenges of the MV modelling.^1^, ^14^, ^177^

Normal tissue stress is maintained in a homoeostatic state.^178^ Changes of the mechanical environment under pathological conditions lead to tissue remodelling, weakening of the structure integrity, and decreased functionality.^179^ MV intervention (e.g., surgical repair) will inevitably alter mechanical environments of the MV apparatus and ventricles. How such changes in mechanical environments influence the long‐term durability of a repaired MV and ventricular pump function is unknown.

Restoring normal tissue homoeostasis (informed by stress prediction of patient‐specific models) should be used to inform MV intervention. Indeed, computational models have already been used to quantify tissue stress with various interventions.^45^, ^46^, ^47^, ^48^ Evaluations of the biomechanical responses have been used on the edge‐to‐edge repair^26^, ^49^, ^51^, ^54^, ^63^, ^180^ and the effects of the annular contraction.^50^, ^160^, ^177^ Such computational strategies can provide quantitative biomechanical characteristics on the physiological and pathological valvular and ventricular functions. Prediction of various interventions' outcome can be used to assess potential functional improvement after virtual interventions from biomechanical perspective.^53^

However, clinical translations of the MV models are limited since most of the models are not truly subject specific as one cannot easily measure the patient‐specific MV properties, the chordal structure, or the boundary conditions. With the advancement of imaging modalities and computational techniques, virtual‐stratified evaluation of surgical intervention can be possible in the future. To this end, further research is required to have (1) large cohort studies to identify physiological and pathological tissue properties, geometries, and boundary conditions; (2) reliable and reproducible modelling procedure,^63^ and (c) predictions on the outcome of possible surgical interventions and important mechanical biomarkers.

## 6 CONCLUSION

In this summary, we have reviewed the current MV models in terms of geometry reconstruction, material properties, and model validation and verifications. Although the majority of the current MV models have focused on structural analysis, MV models that include the FSI and LV interaction started to emerge, and we have particularly focused on the development and methodology of such models. We further demonstrate the feasibility of constructing an in vivo MV‐LV model using in‐house developed immersed boundary method with finite element extension, the results are encouraging and compared favourably with in vivo measurements.

Carefully validated modelling of MV disease and its surgical corrections can provide significant insights into mitral diseases and their interplay with cardiac functions. For this purpose, it is important that stress and strain patterns of MV studies are reported to provide a basis for future verfications. Our summary of current literature on stress and strain patterns suggests that this picture is far from complete. Future directions of MV modelling include the model uncertainty, MV‐heart interaction, and multi‐scale and multi‐physics modelling. However, these tasks require the integration of advanced cardiac imaging, data‐driven mathematical models, state‐of‐the‐art scientific computing infrastructure, and purposely designed experimental/clinical measurements.
